# A circRNA–miRNA–mRNA network identification for exploring underlying pathogenesis and therapy strategy of hepatocellular carcinoma

**DOI:** 10.1186/s12967-018-1593-5

**Published:** 2018-08-09

**Authors:** Dan-dan Xiong, Yi-wu Dang, Peng Lin, Dong-yue Wen, Rong-quan He, Dian-zhong Luo, Zhen-bo Feng, Gang Chen

**Affiliations:** 1 Department of Pathology, First Affiliated Hospital of Guangxi Medical University, 6 Shuangyong Road, Nanning, 530021 Guangxi Zhuang Autonomous Region People’s Republic of China; 2 Department of Medical Ultrasonics, First Affiliated Hospital of Guangxi Medical University, 6 Shuangyong Road, Nanning, 530021 Guangxi Zhuang Autonomous Region People’s Republic of China; 3 Department of Medical Oncology, First Affiliated Hospital of Guangxi Medical University, 6 Shuangyong Road, Nanning, 530021 Guangxi Zhuang Autonomous Region People’s Republic of China

**Keywords:** Hepatocellular carcinoma, CircRNA, ceRNA, CMap

## Abstract

**Background:**

Circular RNAs (circRNAs) have received increasing attention in human tumor research. However, there are still a large number of unknown circRNAs that need to be deciphered. The aim of this study is to unearth novel circRNAs as well as their action mechanisms in hepatocellular carcinoma (HCC).

**Methods:**

A combinative strategy of big data mining, reverse transcription-quantitative polymerase chain reaction (RT-qPCR) and computational biology was employed to dig HCC-related circRNAs and to explore their potential action mechanisms. A connectivity map (CMap) analysis was conducted to identify potential therapeutic agents for HCC.

**Results:**

Six differently expressed circRNAs were obtained from three Gene Expression Omnibus microarray datasets (GSE78520, GSE94508 and GSE97332) using the RobustRankAggreg method. Following the RT-qPCR corroboration, three circRNAs (hsa_circRNA_102166, hsa_circRNA_100291 and hsa_circRNA_104515) were selected for further analysis. miRNA response elements of the three circRNAs were predicted. Five circRNA–miRNA interactions including two circRNAs (hsa_circRNA_104515 and hsa_circRNA_100291) and five miRNAs (hsa-miR-1303, hsa-miR-142-5p, hsa-miR-877-5p, hsa-miR-583 and hsa-miR-1276) were identified. Then, 1424 target genes of the above five miRNAs and 3278 differently expressed genes (DEGs) on HCC were collected. By intersecting the miRNA target genes and the DEGs, we acquired 172 overlapped genes. A protein–protein interaction network based on the 172 genes was established, with seven hubgenes (JUN, MYCN, AR, ESR1, FOXO1, IGF1 and CD34) determined from the network. The Gene Oncology, Kyoto Encyclopedia of Genes and Genomes and Reactome enrichment analyses revealed that the seven hubgenes were linked with some cancer-related biological functions and pathways. Additionally, three bioactive chemicals (decitabine, BW-B70C and gefitinib) based on the seven hubgenes were identified as therapeutic options for HCC by the CMap analysis.

**Conclusions:**

Our study provides a novel insight into the pathogenesis and therapy of HCC from the circRNA–miRNA–mRNA network view.

## Background

Circular RNA (circRNA), with a complete closed loop structure, is firstly identified in 1976 [1]. However, these transcripts without poly-A tail are ignored for a long time due to the limitation of traditional RNA detection methods. In recent years, with the development of high-throughput sequencing technology, a myriad of circRNAs has been found in the eukaryotic transcriptome [2]. With the features of cell-type specific [3] and highly conserved across species [4], circRNAs are thought to be new star RNAs which play important roles in various diseases, including human cancers [5, 6].

Competing endogenous RNAs (ceRNAs) are transcripts that act as miRNA sponges, modulating each other at post-transcriptional level via competely binding to shared miRNAs [7]. Recently, circRNAs have become new hotspots in ceRNA family since they have been demonstrated to harbor abundant conserved miRNA response elements (MREs) [8]. Increasing study has revealed that some circRNAs are involved in tumor initiation and progression by the ceRNA mechanism. For example, a classic cirRNA, ciRs7, is deemed to be a miRNA sponge, absorbing miR-7 and liberating the latter inhibitory effect on its target gene in many human cancers [9, 10]. CircRNAs as ceRNAs mediating pathological processes has also been reported in HCC [11, 12]. However, many unknown circRNAs still remain to be explored.

**Fig. 1:**
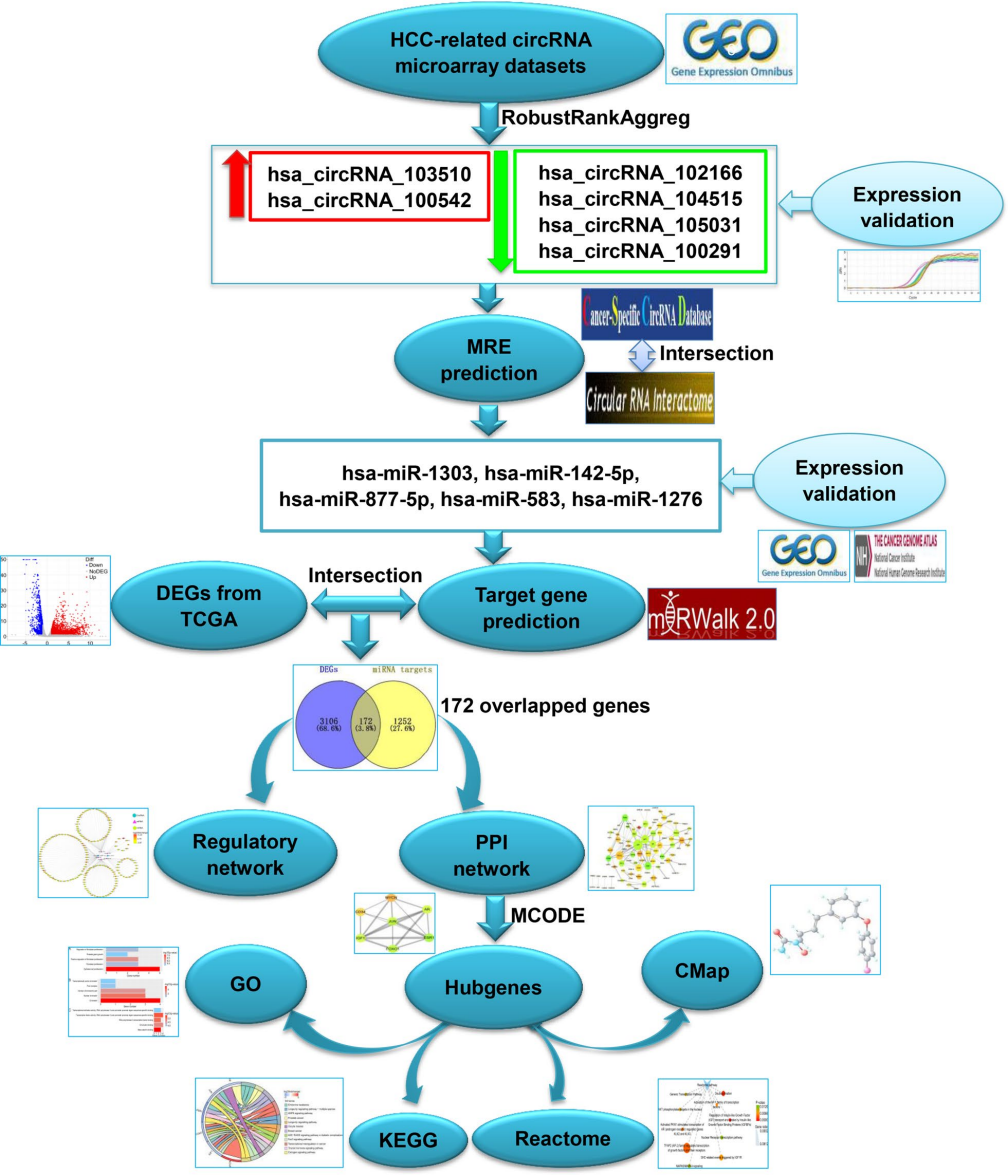
Flow chart of the present study. *HCC* hepatocellular carcinoma, *GEO* Gene Expression Omnibus, *MRE* miRNA response element, *DEGs* differently expressed genes, *TCGA* The Cancer Genome Atlas, *PPI* protein–protein interaction, *MCODE* Molecular Complex Detection, *GO* Gene Oncology, *KEGG* Kyoto Encyclopedia of Genes and Genomes, *CMap* connectivity map

In this study, we employed a combinative strategy of gene chip and computational biology to investigate novel circRNAs and their potential action mechanisms in HCC. The flow chart recapitulating the present work is shown in Fig. 1 as follows: First, we collected HCC-related microarray datasets providing expression profile of circRNAs from the Gene Expression Omnibus (GEO), obtaining differently expressed circRNAs (DECs) with RobustRankAggreg method and corroborating their expression using reverse transcription-quantitative polymerase chain reaction (RT-qPCR). To depict whether the DECs function as ceRNAs in HCC, we collected their sponge miRNAs and miRNA target genes, constructing a circRNA–miRNA–mRNA network. A protein–protein interaction (PPI) network was subsequently established and the hubgenes were identified. Then, Gene Oncology (GO), Kyoto Encyclopedia of Genes and Genomes (KEGG) and Reactome enrichment analyses on the hubgenes were performed to elucidate the potential pathogenesis of HCC. Furthermore, we conducted a connectivity map (CMap) analysis to acquire bioactive compounds for the treatment of HCC, which provide a new insight into the latent therapeutic capacity of circRNAs in HCC.

## Methods

### Screening of DECs in HCC from GEO

Microarray datasets providing circRNA expression profile in HCC were achieved from the GEO database [13]. All raw expression data were normalized and log2-transformed. First, we used Limma, a Bioconductor package for differential analysis of microarray data, to determine DECs in each dataset with the criteria of |log2(foldchange)| > 1 and P-value < 0.05 [14]. Then, we integrated and ranked all of the DECs with R package RobustRankAggreg [15].

### Validation of DECs with RT-qPCR method

Sixteen paired fresh frozen HCC tissues and corresponding adjacent non-tumor tissues were obtained from patients diagnosed with HCC at the First Affiliated Hospital of Guangxi Medical University (Nanning, Guangxi, People’s Republic of China). No patient received any radiotherapy or chemotherapy before surgery. The Ethics Committee of the First Affiliated Hospital of Guangxi Medical University approved this study.

Total RNA was isolated with TRIzol^®^ Reagent (Life technologies, Thermo Fisher Scientific, USA) following the manufacturer’s instruction. Then, 1 µg total RNA was reversed into 20 µl complementary DNA (cDNA) with Geneseed^®^ II First Strand cDNA Synthesis Kit (Geneseed, Guangzhou, China). RT-qPCR was conducted using Geneseed^®^ qPCR SYBR^®^ Green Master Mix (Geneseed) on ABI7500 system (Applied Biosystems, CA, USA) in line with the manufacturer’s procedure. Beta-actin (Geneseed) was set as the endogenous reference. All of the primer sequences used in this study were synthesized by Geneseed and are displayed in Table 1. CircRNA expression was determined using the 2^−ΔCT^ method. Significance between groups was analyzed by paired T-test with SPSS 22.0 (IBM, New York, USA). A P-value < 0.05 denotes a statistical significance.

**Table 1:** Primer sequences for reverse transcription-quantitative polymerase chain reaction

Gene ID	Primer sequence		Product length (bp)
	Forward (5′-3′)	Reverse (5′-3′)	
ACTB	CATGTACGTTGCTATCCAGGC	CTCCTTAATGTCACGCACGAT	250
hsa_circRNA_102166	TACGTTGATCACCAAGGGCT	CTTCTGCTTTGGCTGTGACA	126
hsa_circRNA_104515	CTTTATAACTATAGGGTACTGG	GTCTCCTCTGGTTCATTG	150
hsa_circRNA_105031	ACTACAGGCAATCAGGGTTC	GTACAAGTTCTGCAGGAACGA	118
hsa_circRNA_100291	CATTCTTATAGTTGTAAGCTTAG	CATAGGAGAAAGCATCATTAT	131

*ACTB* beta-actin

### Prediction of MREs

The miRNA binding sites, also known as MREs, of those selected DECs, were predicted with two web tools, Cancer-Specific CircRNA (CSCD) [16] and Circular RNA Interactome (CircInteractome) [17]. We identified overlapped miRNAs of the two algorithms as potential target miRNAs of the DECs.

### Verification of miRNA expression based on data from GEO and TCGA

Microarray datasets providing miRNA expression profile in HCC were obtained from the public databases GEO and The Cancer Genome Atlas (TCGA) [18]. The retrieval terms were as follows: (hepatocellular OR hepatic OR liver OR HCC) and (“cancer” OR “tumor” OR “tumour” OR “carcinoma” OR “neoplasm” OR “malignan*”) and (miRNA OR microRNA OR miR OR “non-coding RNA” OR ncRNA OR “noncoding RNA” OR “non coding RNA”).

We screened available datasets based on the listed inclusion criteria: (1) all of the patients were diagnosed with HCC; (2) the studies must contain circRNA expression data both in cancerous and normal liver tissues; and (3) the sample sizes in tumor and non-tumor group were at least three.

Two revivers extracted the basic information of each included record: miRNA type, first author and published year, region, data source, platform, number of case, and expression level of miRNA. Any divergences were settled via discussion with a third investigator.

The combined standard mean difference (SMD) and 95% confidence interval (95% CI) were computed by STATA 12.0 (StataCorp, College Station, TX, USA). A SMD > 0 represents high expression of miRNA in HCC samples than in normal controls. The corresponding 95% CI do not cross 1 and a P-value < 0.05 suggest a statistical significance.

### Prediction of miRNA target genes

The miRNA–mRNA interactions were predicted with miRWalk 2.0 [19], which involves 12 predicted algorithms (Targetscan, RNAhybrid, RNA22, PITA, Pictar2, miRWalk, Microt4, miRNAMap, miRDB, mirbridge, miRanda and miRMap). Target genes forecasted by at least eight algorithms were selected for further analysis.

### Collection of differently expressed genes (DEGs) of HCC form TCGA

RNA-sequencing (RNA-seq) data containing 374 HCC samples and 50 normal controls was downloaded from the TCGA. DEGs were determined by the edgeR package [20] in Bioconductor with the filter criteria of |log2(foldchange)| > 1 and adjust P-value < 0.05.

### Construction of circRNA–miRNA–mRNA network

The overlapping genes between the predicted miRNA target genes and the DEGs were obtained for circRNA–miRNA–mRNA network construction. The Cytoscape 3.6.1 software [21] was used to visualize the regulatory network.

### Establishment of PPI network and identification of hub-genes

A PPI network was established by the STRING (v10.5) [27924014] and visualized by the Cytoscape 3.6.1. Then, the “Molecular Complex Detection” (MCODE), a clustering algorithm identifying locally densely connected regions in a large PPI network based on a node-weighting arithmetic [22], was employed to recognize highly interacted hubgene clustering.

### GO, KEGG and Reactome enrichment analyses

GO annotation and KEGG pathway analyses were conducted by clusterProfiler, an R package for functional classification and enrichment of gene clusters using hypergeometric distribution [23]. Reactome pathway analysis was performed by Reactome FI, a plugin of Cytoscape for network and pathway analysis [24].

### CMap analysis

Hubgenes consisting of two lists of up- and down-regulated tags were uploaded into the CMap web tool, matching against over 7000 gene expression profiles following treatment of 1309 bioactive compounds in human cell lines [25]. The match between the signatures of interest and chemicals from CMap was assessed by a connectivity score from − 1 to 1: a positive score denotes a stimulative effect of compound on the query signatures; while a negative score implicates a repressed effect of a compound on the query signatures.

## Results

### Identification of six DECs in HCC based on RobustRankAggreg method

**Fig. 2:**
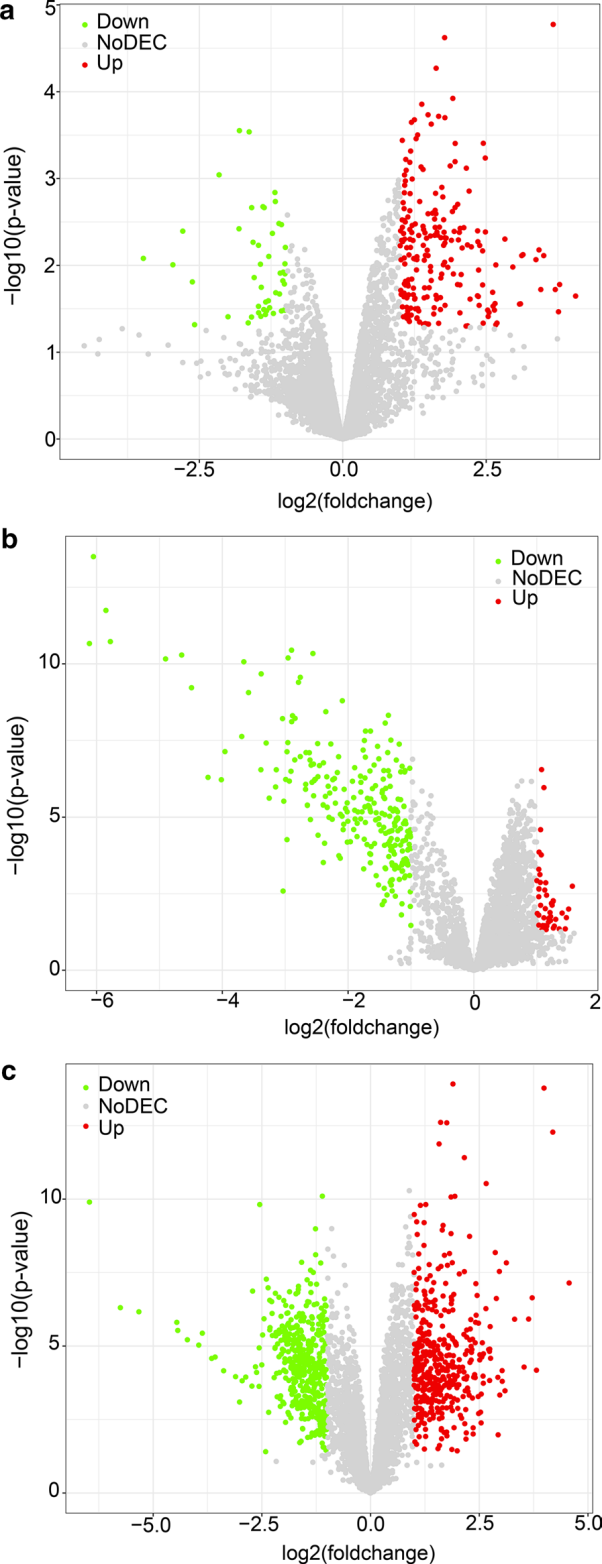
Volcano plots for DECs in HCC based on the three microarray datasets from GEO: **a** GSE78520, **b** GSE94508, **c** GSE97332. The volcano plot was generated by R package ‘ggplot2’. *DECs* differently expressed circRNAs, *HCC* hepatocellular carcinoma, *GEO* Gene Expression Omnibus

**Fig. 3:**
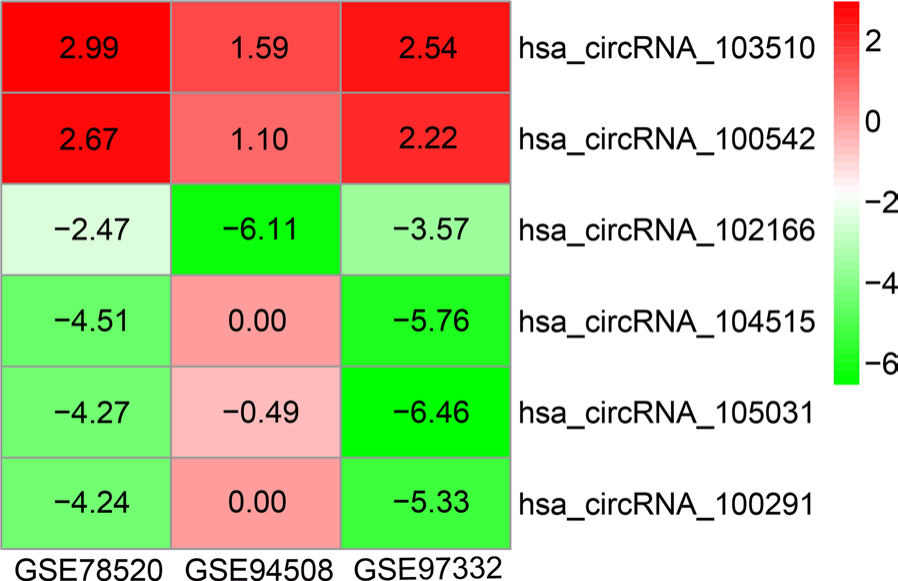
Heatmap for the six DECs determined using the RobustRankAggreg method with an adjust P-value < 0.05. The heatmap was generated by R package ‘pheatmap’. *DECs* differently expressed circRNAs

**Fig. 4:**
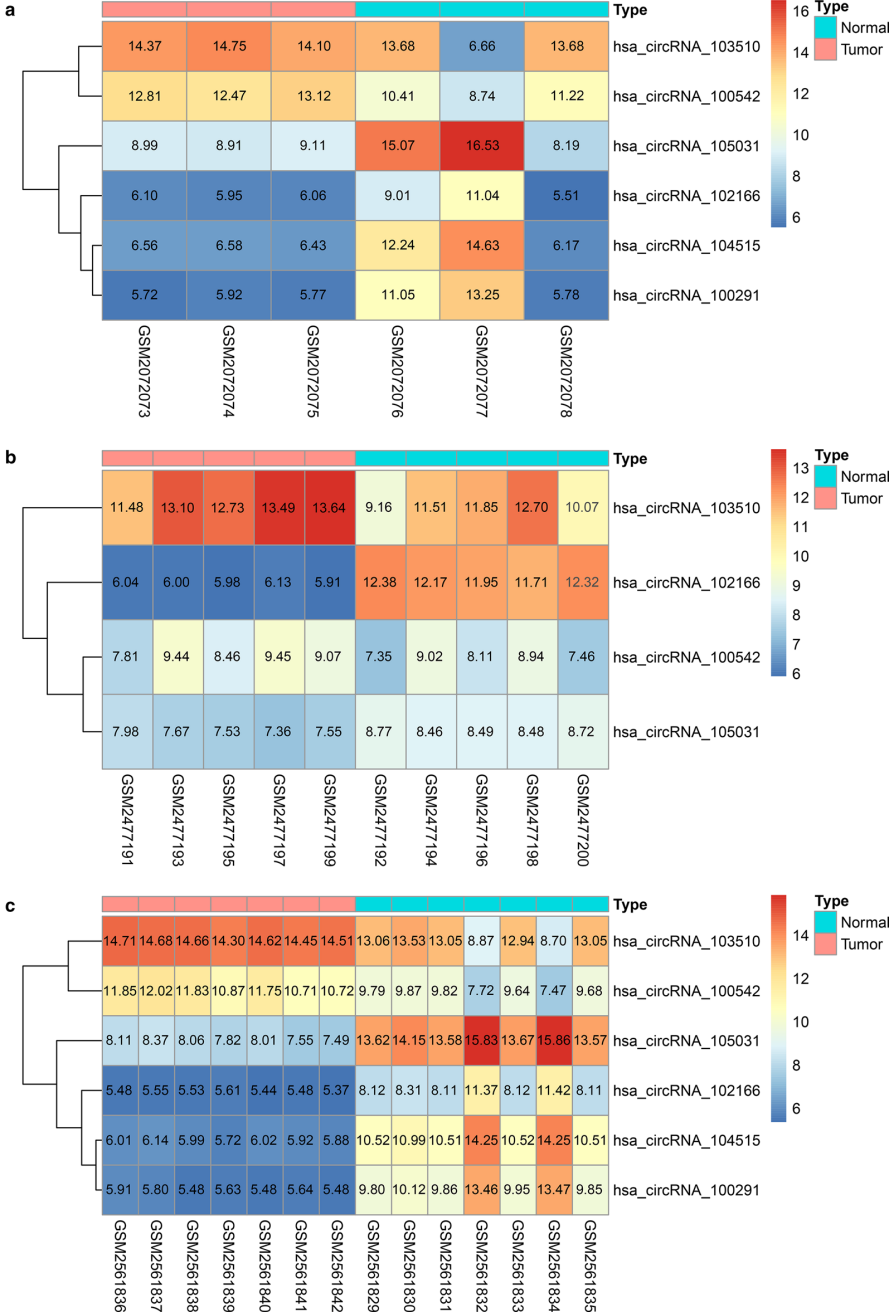
Heatmap for the six DECs in individual microarray datasets: **a** GSE78520, **b** GSE94508, **c** GSE97332. The heatmap was generated by R package ‘pheatmap’. *DECs* differently expressed circRNAs

**Fig. 5:**
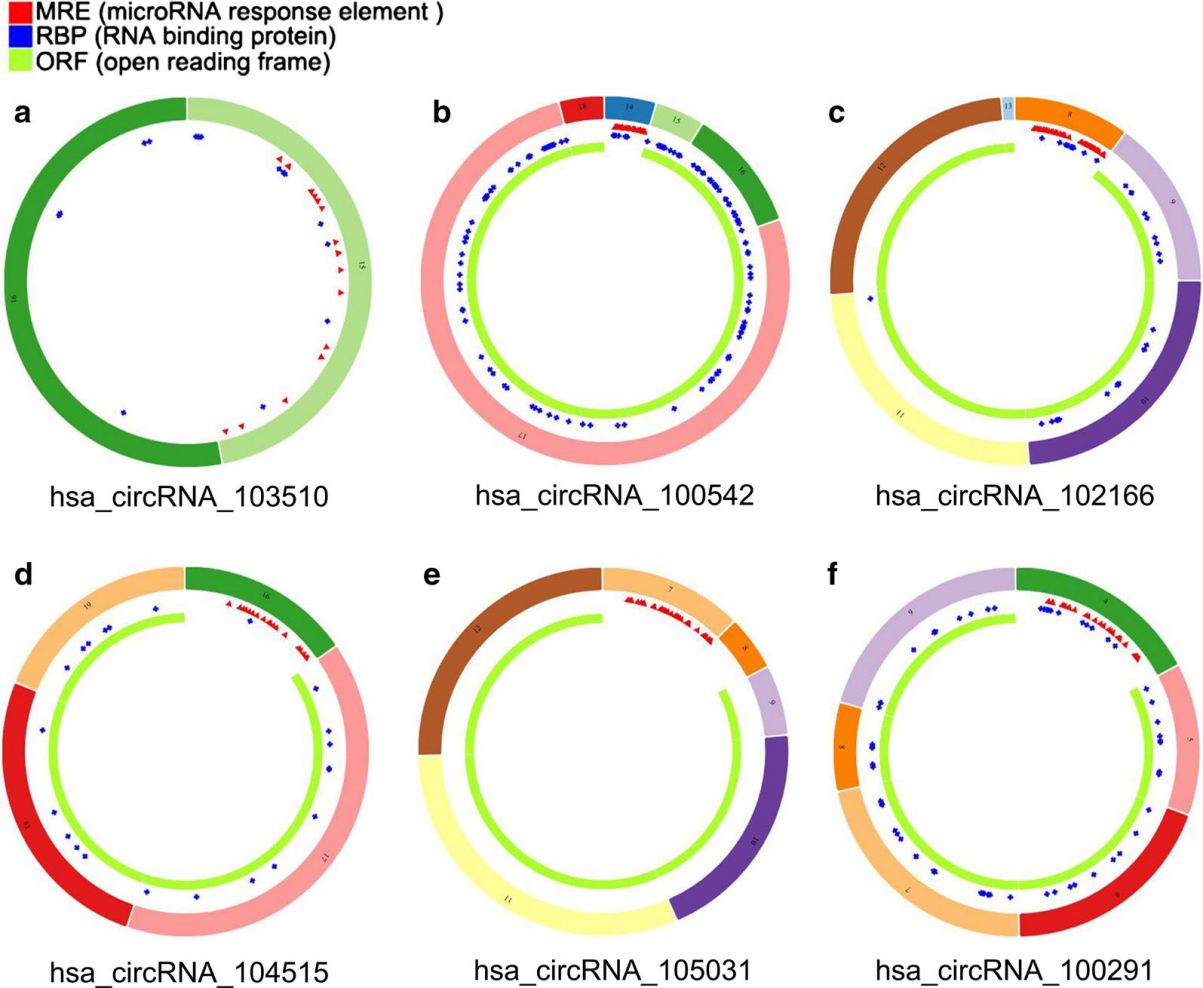
Structural patterns of the six circRNAs by the Cancer-Specific CircRNA (CSCD, http://gb.whu.edu.cn/CSCD/#): **a** has_circRNA_103510, **b** has_circRNA_100542, **c** has_circRNA_102166, **d** has_circRNA_104515, **e** has_circRNA_105031, **f** has_circRNA_100291

Three microarray datasets (GSE78520, GSE94508 and GSE97332) were included in our study. All of the three gene chips were from the platform of Agilent-069978 Arraystar Human CircRNA microarray V1. The basic information of the three datasets is concluded in Table 2. A total of 259 DECs with 211 up-regulated circRNAs and 48 down-regulated circRNAs were found in gene chip GSE78520 (Fig. 2a); 299 DECs with 46 up-regulated circRNAs and 253 down-regulated circRNAs were determined in gene chip GSE94508 (Fig. 2b); 882 DECs with 429 up-regulated circRNAs and 453 down-regulated circRNAs were identified in gene chip GSE97332 (Fig. 2c). Subsequently, we integrated the DECs of the three datasets and ranked them with a robust method. A total of six circRNAs, including two up-regulated circRNAs (hsa_circRNA_103510 and hsa_circRNA_100542) and four down-regulated circRNAs (hsa_circRNA_102166, hsa_circRNA_104515, hsa_circRNA_105031 and hsa_circRNA_100291), were found to be in the top rankings with an adjust P-value < 0.05 (Fig. 3). The expression of the six circRNAs in each dataset is shown in Fig. 4. The essential characteristics of the six circRNAs are displayed in Table 3. The basic structural patterns of the six circRNAs are exhibited in Fig. 5.

**Table 2:** Basic information of the three microarray datasets from GEO

Data source	Platform	First author	Year	Region	Sample size (T/N)	Number of circRNAs
GSE78520	GPL19978	Li C	2016	China	3/3	4451
GSE94508	GPL19978	Fu L	2017	China	5/5	2572
GSE97332	GPL19978	Han D	2017	China	7/7	3471

*GEO* Gene Expression Omnibus, *T* tumor, *N* normal

**Table 3:** Essential characteristics of the six differently expressed circRNAs

CircRNA	Alias	CircRNA type	Position	Strand	Best transcript	Gene symbol	Regulation
hsa_circRNA_103510	hsa_circ_0067934	Exonic	chr3:170013698-170015181	+	uc003fgs.2	PRKCI	Up
hsa_circRNA_100542	hsa_circ_0017639	Exonic	chr10:7290509-7327916	−	uc010qay.2	SFMBT2	Up
hsa_circRNA_102166	hsa_circ_0004913	Exonic	chr17:62248459-62265775	−	uc002jec.3	TEX2	Down
hsa_circRNA_104515	hsa_circ_0002980	Exonic	chr7:141336759-141349133	+	uc003vwi.2	AGK	Down
hsa_circRNA_105031	hsa_circ_0091570	Exonic	chrX:131516205-131526362	−	uc004ewt.3	MBNL3	Down
hsa_circRNA_100291	hsa_circ_0000098	Exonic	chr1:101372407-101387397	+	uc001dtn.2	SLC30A7	Down

### Corroboration of the six circRNAs with RT-qPCR

**Fig. 6:**
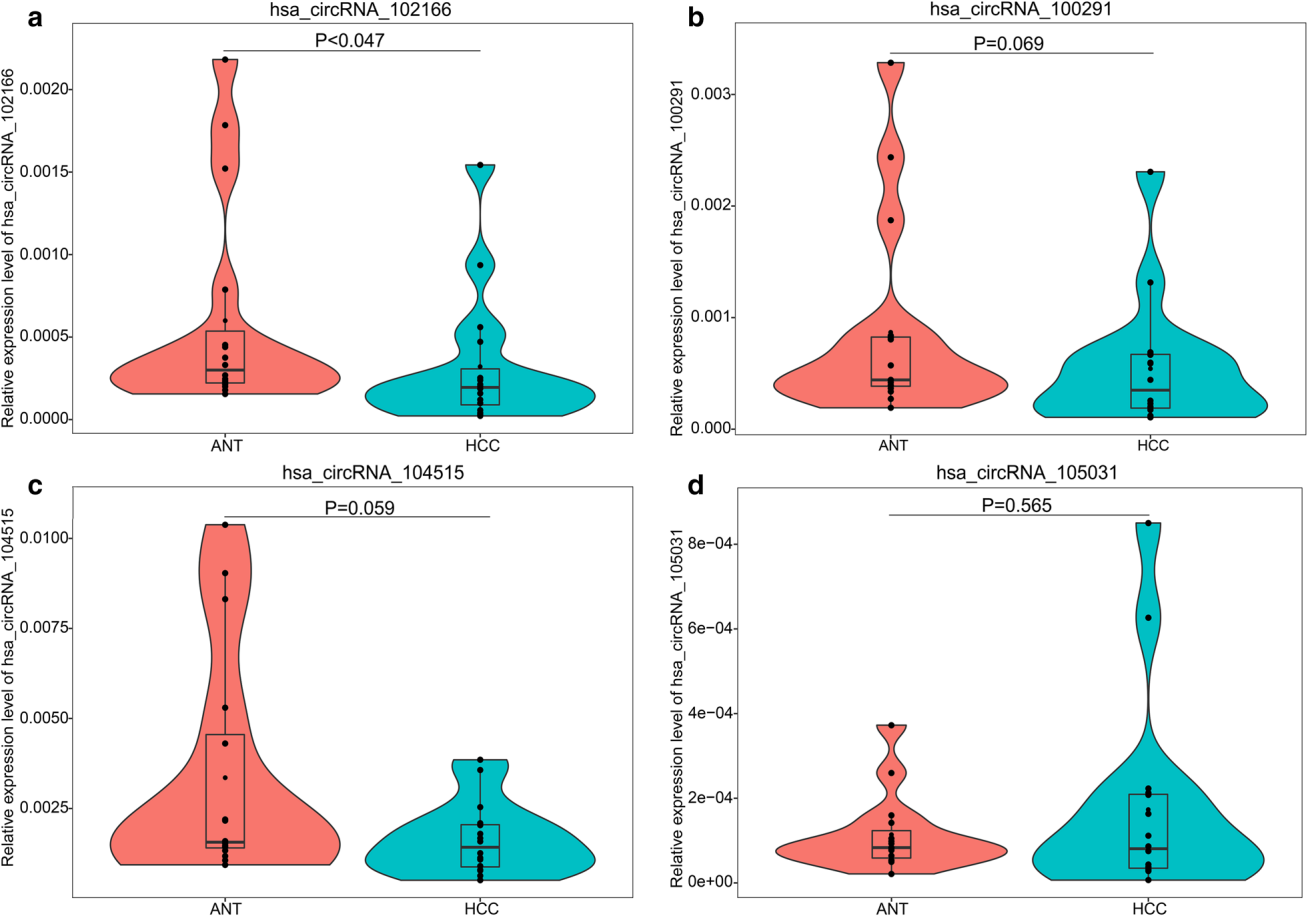
Violin plots for the expression of hsa_circRNA_102166, hsa_circRNA_104515, hsa_circRNA_105031 and hsa_circRNA_100291 in HCC by RT-qPCR: **a** hsa_circRNA_102166, **b** hsa_circRNA_100291, **c** hsa_circRNA_104515, **d** hsa_circRNA_105031. *HCC* hepatocellular carcinoma, *ANT* adjacent non-tumorous, *RT-qPCR* reverse transcription-quantitative polymerase chain reaction

**Fig. 7:**
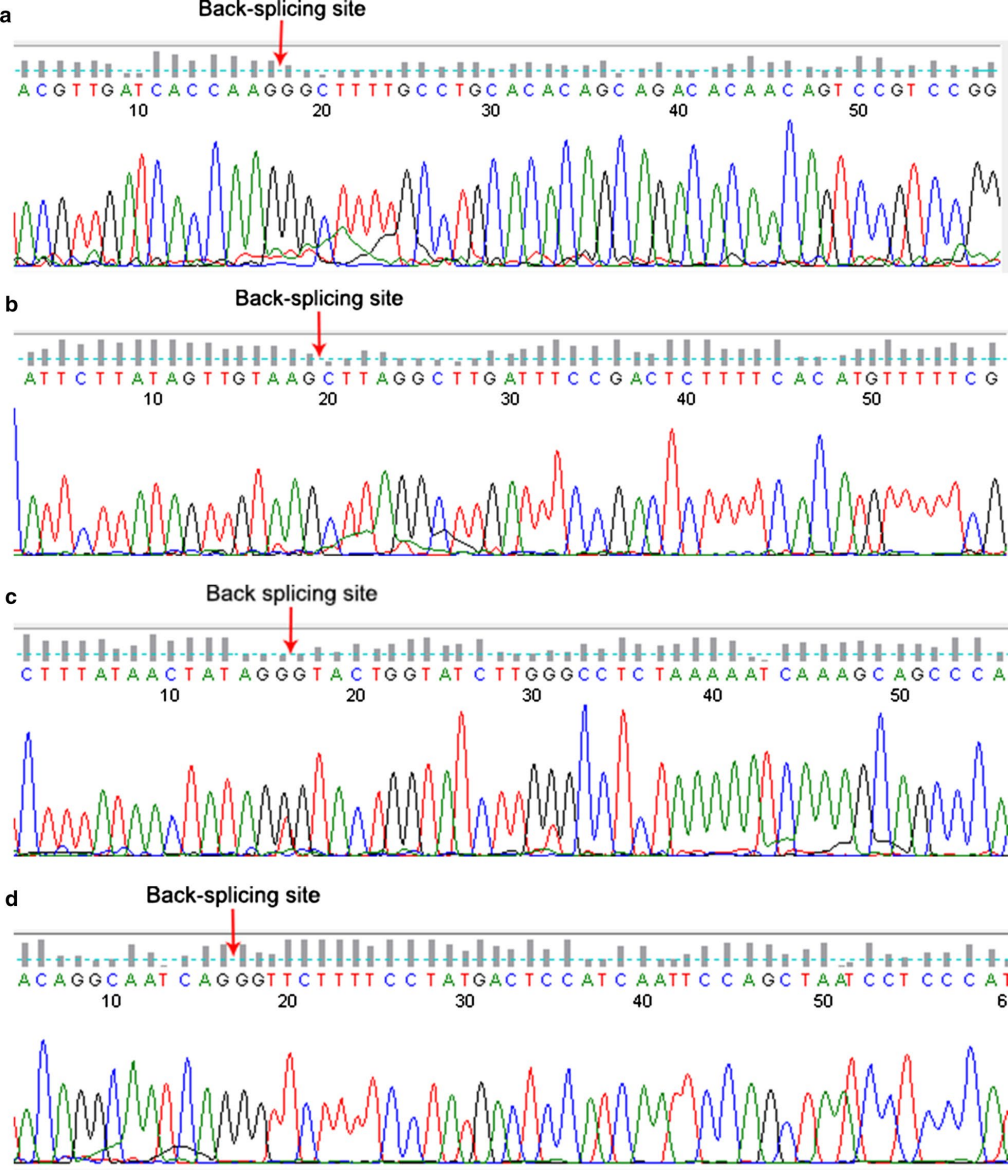
Head-to-tail splicing in the RT-qPCR product of hsa_circRNA_102166, hsa_circRNA_100291, hsa_circRNA_104515 and hsa_circRNA_105031 by Sanger sequencing: **a** hsa_circRNA_102166, **b** hsa_circRNA_100291, **c** hsa_circRNA_104515, **d** hsa_circRNA_105031

RT-qPCR was conducted in 16 pairs HCC samples and adjacent non-cancerous tissues to corroborate the expression of the six circRNAs. Among the six circRNAs, hsa_circRNA_103510 and hsa_circRNA_100542 cannot be detected in these in-house tissues due to their relative low expression levels. The low expression of hsa_circRNA_102166 in HCC tissues was corroborated by RT-qPCR (P = 0.047, Fig. 6a). Hsa_circRNA_100291 and hsa_circRNA_104515 showed a down-regulated tendency in HCC, though the P-values were greater than 0.05 (hsa_circRNA_100291: P = 0.069, Fig. 6b; hsa_circRNA_104515: P = 0.059, Fig. 6c). For hsa_circRNA_105031, its expression in HCC tissues was similar to that in non-cancerous tissues (P = 0.588, Fig. 6d). The head-to-tail splicing in the RT-qPCR product of hsa_circRNA_102166, hsa_circRNA_100291, hsa_circRNA_104515 and hsa_circRNA_105031 was confirmed by Sanger sequencing (Fig. 7).

### Identification of five circRNA–miRNA interactions

**Fig. 8:**
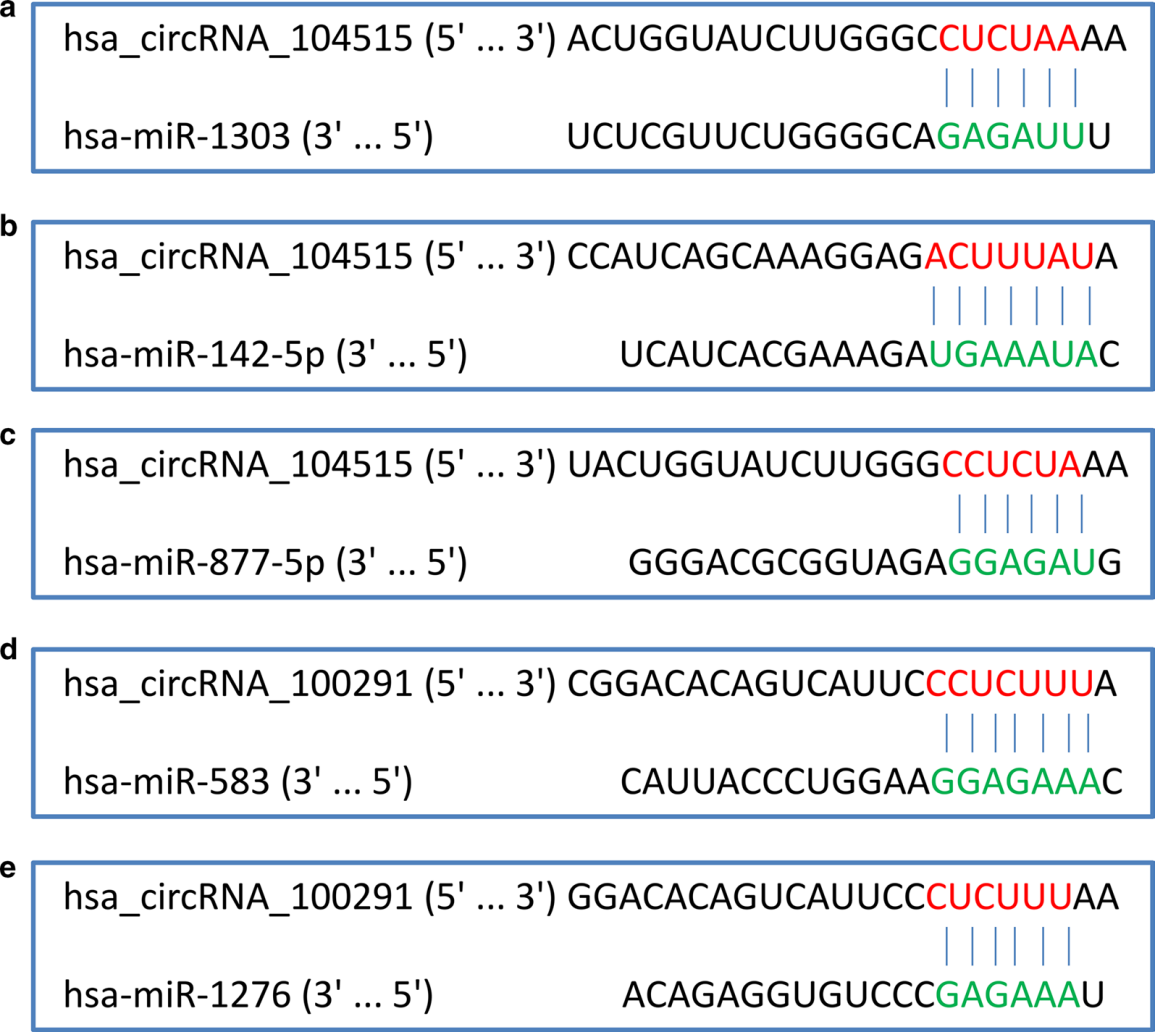
CircRNA–miRNA interactions identified by both databases of CSCD (http://gb.whu.edu.cn/CSCD/#) and CircInteractome (https://circinteractome.nia.nih.gov/index.html): **a** hsa_circRNA_104515/hsa-miR-1303, **b** hsa_circRNA_104515/hsa-miR-142-5p, **c** hsa_circRNA_104515/hsa-miR-877-5p, **d** hsa_circRNA_100291/hsa-miR-583, **e** hsa_circRNA_100291/hsa-miR-1276. CSCD: Cancer-Specific CircRNA; CircInteractome: Circular RNA Interactome

**Fig. 9:**
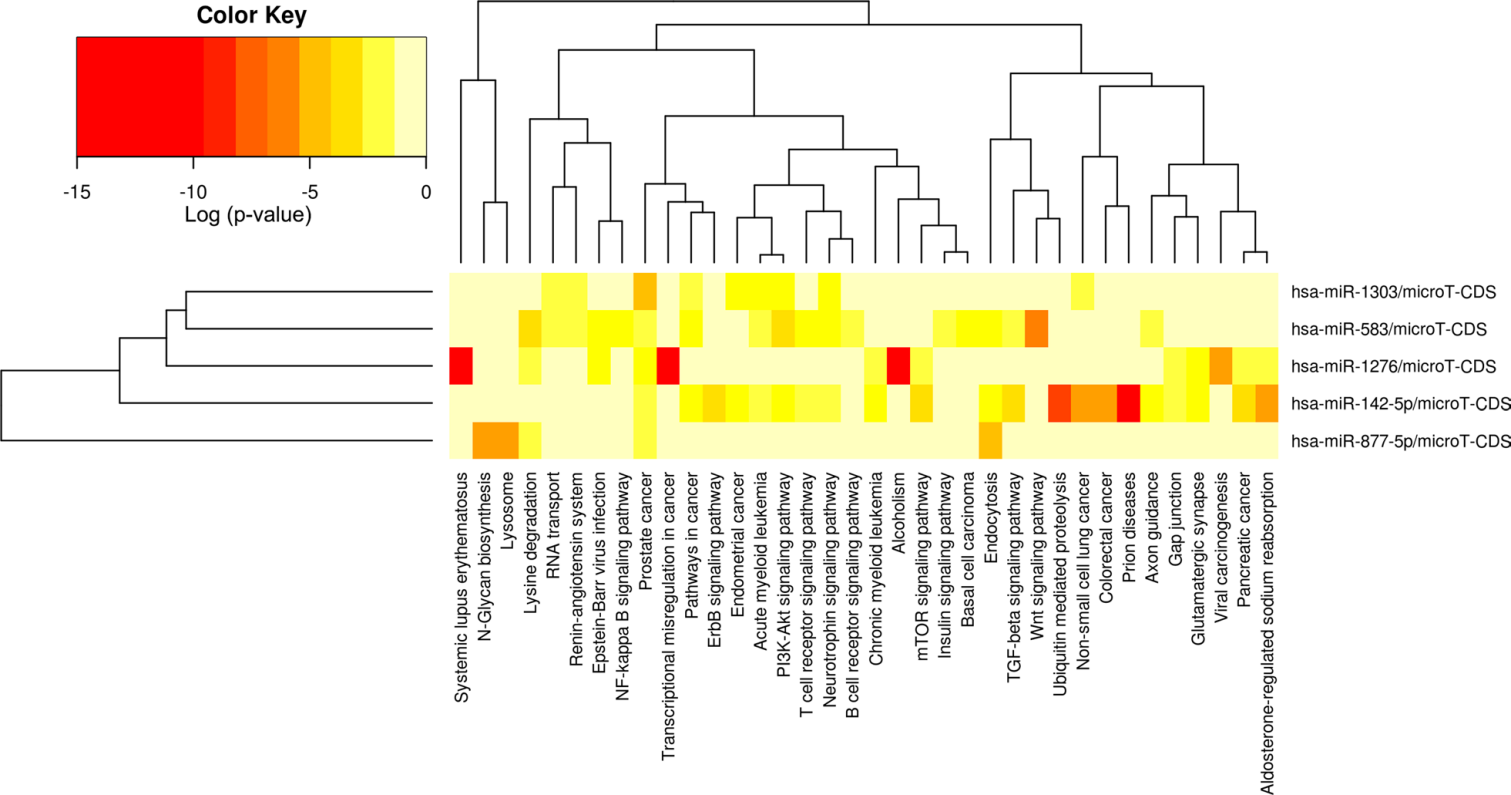
Heatmap for the significant signaling pathways that the five miRNAs mediate according to the DIANA-miRPath

Hsa_circRNA_102166, hsa_circRNA_100291 and hsa_circRNA_104515 were selected for further analysis. Increasing evidence has demonstrated that some circRNAs play critical roles in tumors by functioning as “decoys” to sponge miRNAs. To depict whether the three circRNAs perform the similar role in HCC, we collected their potential target miRNAs from two online databases, CSCD and CircInteractome. A total of five circRNA–miRNA interactions including two circRNAs (hsa_circRNA_104515 and hsa_circRNA_100291) and five miRNAs (hsa-miR-1303, hsa-miR-142-5p, hsa-miR-877-5p, hsa-miR-583 and hsa-miR-1276) were identified (Fig. 8). DIANA-miRPath [26] was exploited to explore the signaling pathways in which the five miRNAs may be involved. As shown in Fig. 9, all of the five miRNAs were closely linked with some cancer-related pathways.

### Expression of the five miRNAs based on the data from GEO and TCGA

**Fig. 10:**
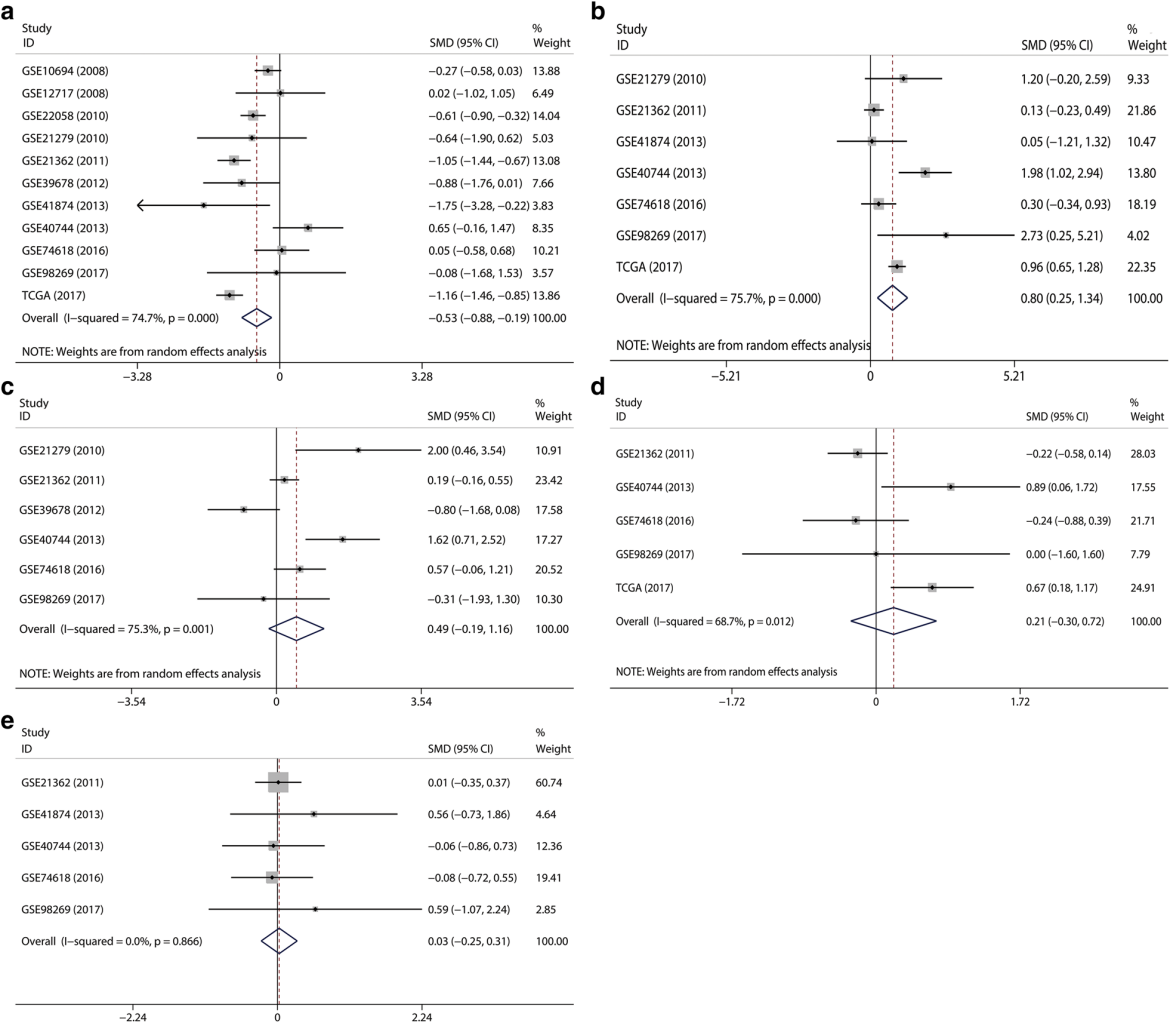
Forest plots of datasets evaluating the expression of the five miRNAs in hepatocellular carcinoma: **a** hsa-miR-142-5p, **b** hsa-miR-877-5p, **c** hsa-miR-1303, **d** hsa-miR-1276, **e** hsa-miR-583

In all, 11 microarray and RNA-seq datasets were included. Among them, 7 datasets with 339 HCC samples and 113 normal controls were for miR-1303; 11 datasets with 881 HCC samples and 351 normal controls were for miR-142-5p; 7 datasets with 671 HCC samples and 149 normal controls were for miR-877-5p; 5 datasets with 307 HCC samples and 97 normal controls were for miR-583; and 5 datasets with 534 HCC samples and 110 normal controls were for miR-1276. The essential properties of the 11 records are concluded in Table 4. According to the pooled results, miR-142-5p was down-regulated (SMD = − 0.53, 95% CI − 0.88  to  − 0.19, P = 0.003; Fig. 10a) and miR-877 was up-regulated (SMD = 0.80, 95% CI 0.25–1.34, P = 0.004; Fig. 10b) in HCC. MiR-1303 and miR-1276 showed high expression tendencies in HCC tissues compared to in normal liver tissues, while the P-values were greater than 0.05 (miR-1303: SMD = 0.49, 95% CI − 0.19  to 1.16, P = 0.157; Fig. 10c; miR-1276: SMD = 0.21, 95% CI − 0.30 to 0.72, P = 0.422; Fig. 10d). For miR-583, its expression in HCC tissues was similar to that in normal liver tissues (SMD = 0.03, 95% CI − 0.25 to 0.31, P = 0.851, Fig. 10e).

**Table 4:** Essential properties of the 11 records for the five miRNAs by data from GEO

miRNA	First author	Year	Region	Data source	Platform	Number of case		Expression (mean ± SD)	
						Cancer	Normal	Cancer	Normal
hsa-miR-1303	Hou J	2010	China	GSE21279	GPL9052	4	7	4.15 ± 1.206	2.49 ± 0.554
	Sato F	2011	Japan	GSE21362	GPL10312	59	61	1.59 ± 1.014	1.39 ± 1.042
	Kim J	2012	South Korea	GSE39678	GPL15852	16	8	11.00 ± 0.702	11.49 ± 0.364
	Diaz G	2013	USA	GSE40744	GPL14613	9	19	3.72 ± 0.774	2.78 ± 0.492
	Villanueva A	2016	Spain	GSE74618	GPL14613	230	10	1.88 ± 0.658	1.51 ± 0.263
	TCGA	2017	USA	TCGA	none	21	5	0.35 ± 0.225	0.17 ± 0.100
	Xie Z	2017	China	GSE98269	GPL20712	3	3	5.07 ± 0.071	5.10 ± 0.115
hsa-miR-142-5p	Li W	2008	China	GSE10694	GPL6542	78	88	11.01 ± 0.605	11.19 ± 0.710
	Su H	2008	China	GSE12717	GPL7274	9	6	9.11 ± 1.406	9.09 ± 0.707
	Burchard J	2010	USA	GSE22058	GPL10457	96	96	0.95 ± 0.241	1.07 ± 0.135
	Hou J	2010	China	GSE21279	GPL9052	4	7	7.04 ± 1.190	7.77 ± 1.112
	Sato F	2011	Japan	GSE21362	GPL10312	59	61	7.06 ± 1.022	7.93 ± 0.577
	Kim J	2012	South Korea	GSE39678	GPL15852	16	8	11.10 ± 0.959	11.88 ± 0.182
	Morita K	2013	Japan	GSE41874	GPL7722	6	4	0.72 ± 0.412	1.34 ± 0.225
	Diaz G	2013	USA	GSE40744	GPL14613	9	19	1.96 ± 0.506	1.74 ± 0.224
	Villanueva A	2016	Spain	GSE74618	GPL14613	230	10	1.37 ± 0.206	1.36 ± 0.212
	Xie Z	2017	China	GSE98269	GPL20712	3	3	6.76 ± 0.733	6.88 ± 0.169
	TCGA	2017	USA	TCGA	None	371	49	5.45 ± 1.264	6.86 ± 0.796
hsa-miR-877-5p	Hou J	2010	China	GSE21279	GPL9052	4	6	3.64 ± 1.753	2.19 ± 0.713
	Sato F	2011	Japan	GSE21362	GPL10312	59	61	2.17 ± 0.921	2.05 ± 0.961
	Morita K	2013	Japan	GSE41874	GPL7722	6	4	1.08 ± 0.313	1.06 ± 0.452
	Diaz G	2013	USA	GSE40744	GPL14613	9	19	5.36 ± 0.619	3.88 ± 0.797
	Villanueva A	2016	Spain	GSE74618	GPL14613	230	10	3.64 ± 0.741	3.42 ± 0.636
	Xie Z	2017	China	GSE98269	GPL20712	3	3	5.31 ± 0.073	5.12 ± 0.066
	TCGA	2017	USA	TCGA	None	360	46	1.47 ± 0.807	0.73 ± 0.328
hsa-miR-583	Sato F	2011	Japan	GSE21362	GPL10312	59	61	1.47 ± 0.722	1.46 ± 0.837
	Morita K	2013	Japan	GSE41874	GPL7722	6	4	0.91 ± 0.249	0.76 ± 0.293
	Diaz G	2013	USA	GSE40744	GPL14613	9	19	1.75 ± 0.247	1.77 ± 0.344
	Villanueva A	2016	Spain	GSE74618	GPL14613	230	10	1.43 ± 0.237	1.45 ± 0.294
	Xie Z	2017	China	GSE98269	GPL20712	3	3	5.07 ± 0.119	5.02 ± 0.020
hsa-miR-1276	Sato F	2011	Japan	GSE21362	GPL10312	59	61	1.55 ± 0.810	1.72 ± 0.737
	Diaz G	2013	USA	GSE40744	GPL14613	9	19	1.9 ± 0.254	1.70 ± 0.209
	Villanueva A	2016	Spain	GSE74618	GPL14613	230	10	1.34 ± 0.164	1.38 ± 0.191
	Xie Z	2017	China	GSE98269	GPL20712	3	3	5.06 ± 0.049	5.06 ± 0.027
	TCGA	2017	USA	TCGA	None	233	17	0.61 ± 0.380	0.36 ± 0.233

*TCGA* The Cancer Genome Atlas, *SD* standard deviation

### Construction of circRNA–miRNA–mRNA network

**Fig. 11:**
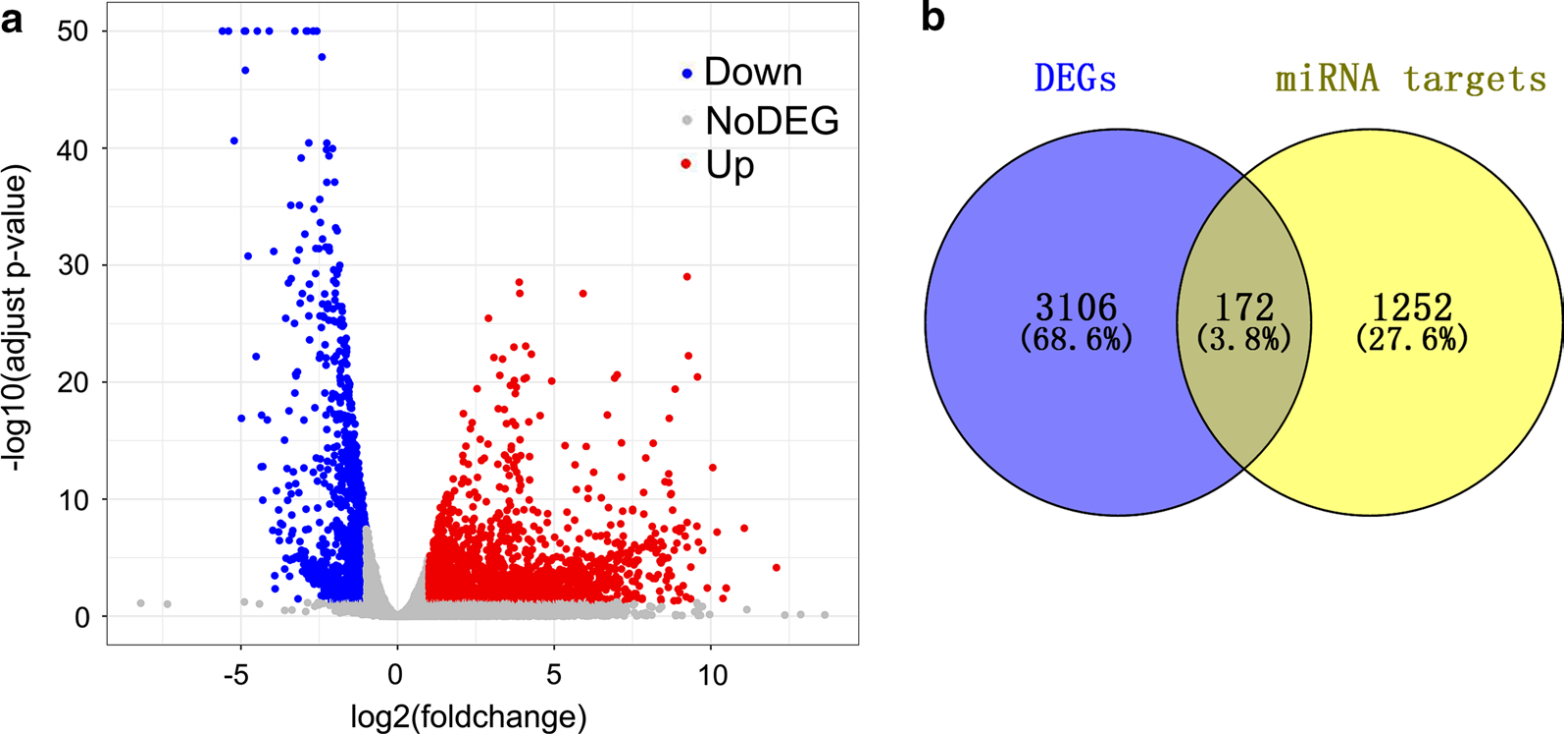
Identification of 172 genes that exert momentous roles in hepatocellular carcinoma (HCC). **a** Volcano plot of the differentially expressed genes (DEGs) in HCC based on data from TCGA. The volcano plot was generated by R package ‘ggplot2’. **b** Venn diagram for the intersections between DEGs and miRNA target genes

Total 1424 target genes of the aforementioned five miRNAs were obtained from the miRWalk. Additionally, 3278 DEGs in HCC were gained from the TCGA (Fig. 11a). By intersecting the predicted target genes and DEGs, we identified 172 target genes that exert momentous roles in HCC (Fig. 11b).

**Fig. 12:**
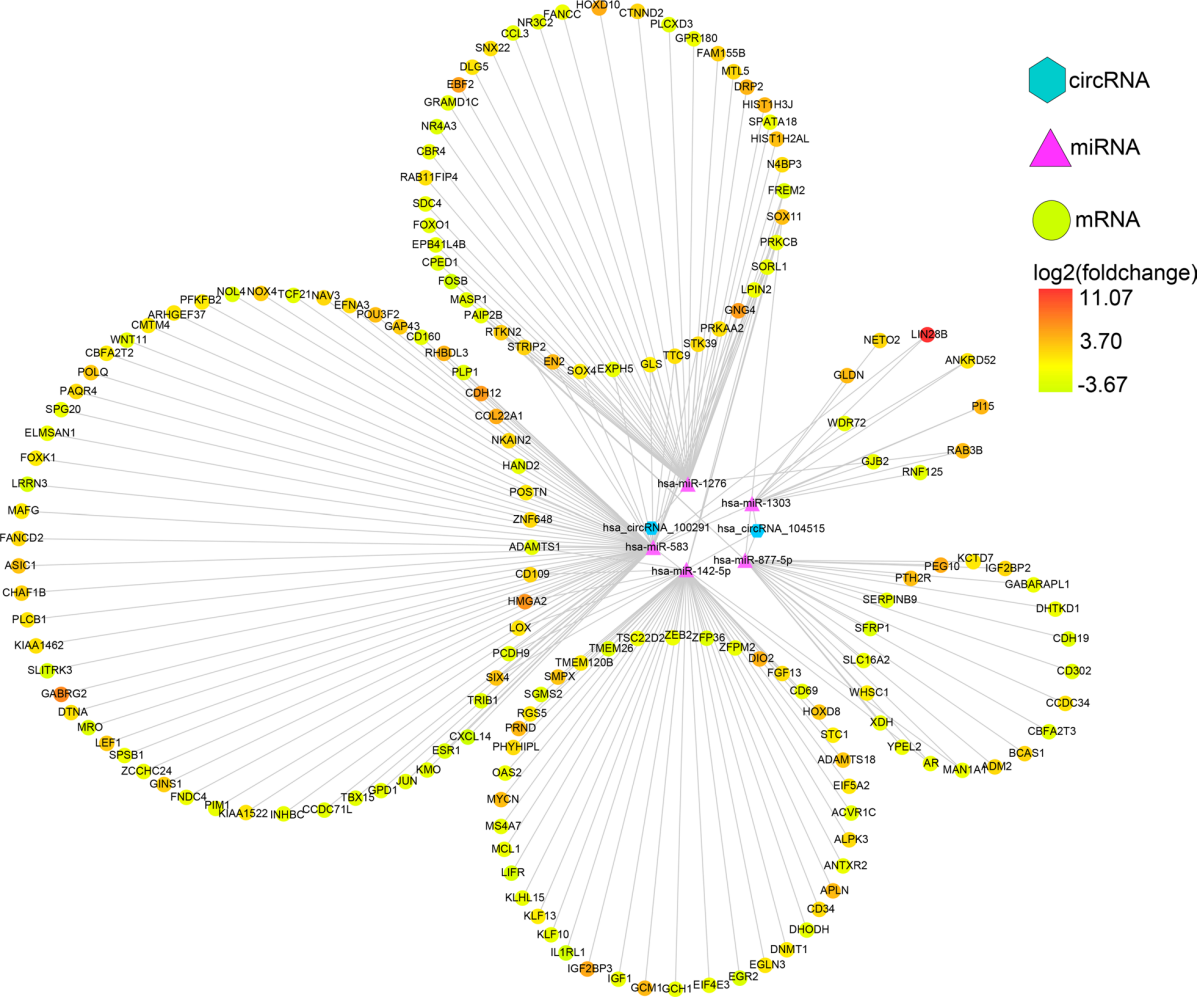
circRNA–miRNA–mRNA regulatory network. The network consisting of two cricRNAs (hsa_circRNA_104515 and hsa_circRNA_100291), five miRNAs (hsa-miR-1303, hsa-miR-142-5p, hsa-miR-877-5p, hsa-miR-583 and hsa-miR-1276) and 172 genes was generated by Cytoscape 3.6.1

We integrated the circRNA–miRNA interactions and miRNA–mRNA interactions to construct a circRNA–miRNA–mRNA network (Fig. 12), which provided a preliminary insight into the links between the DECs (hsa_circRNA_104515 and hsa_circRNA_100291), the five miRNAs (hsa-miR-1303, hsa-miR-142-5p, hsa-miR-877-5p, hsa-miR-583 and hsa-miR-1276) and the 172 mRNAs.

### Identification of seven hubgenes with MCODE algorithm from PPI network

**Fig. 13:**
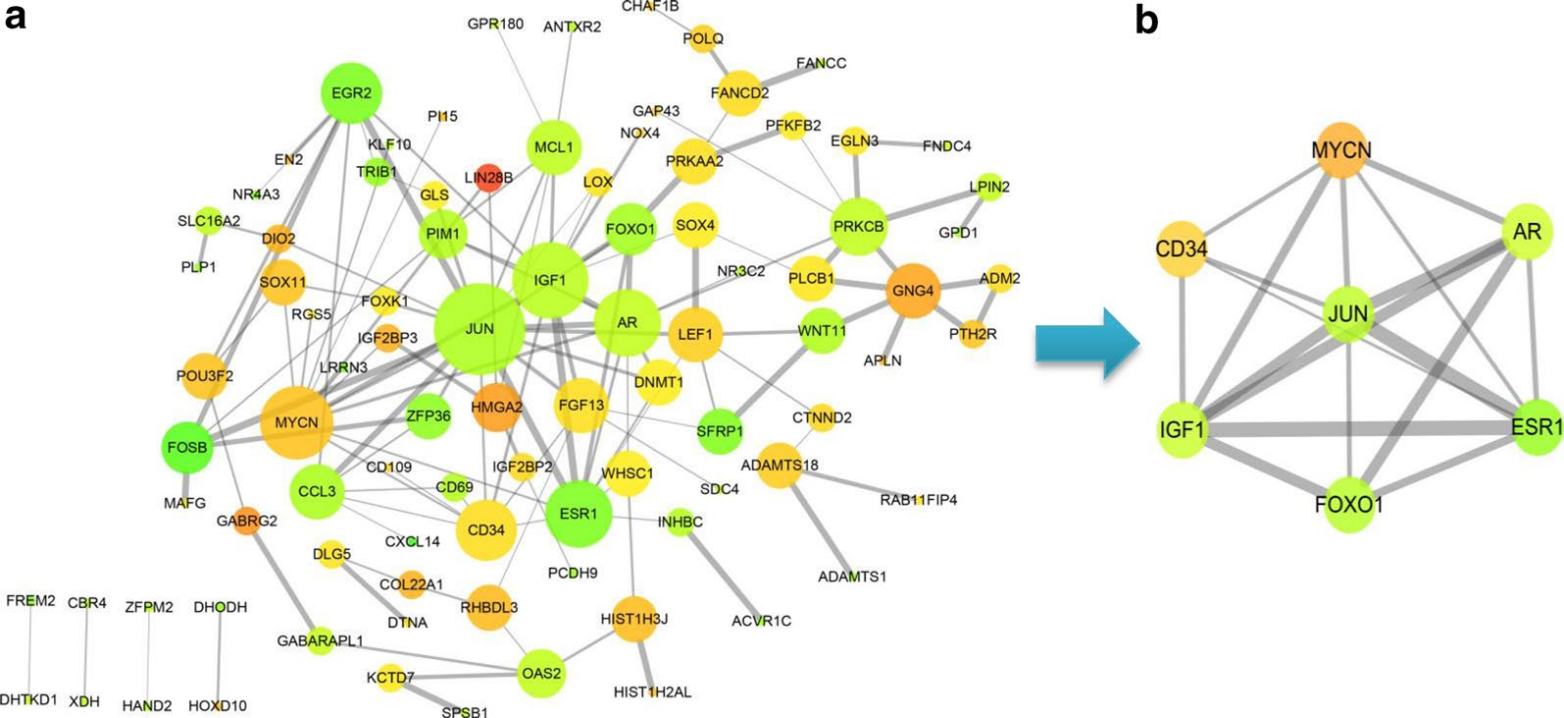
Identification of hubgenes from the PPI network with the MCODE algorithm. The node color changes gradually from green to red in ascending order according to the log2(foldchange) of genes. The edge size changes gradually from fine to coarse in ascending order according to the combined score between two neighbored genes. **a** A PPI network of the 172 target genes that exert momentous roles in hepatocellular carcinoma. This network consists of 91 nodes and 131 edges. The node size changes gradually from small to large in ascending order according to the number of neighbored genes per gene. **b** A PPI network of the 7 hubgenes that extracted from **a**. This network consists of 7 nodes and 18 edges. *PPI* protein–protein interaction, *MCODE* Molecular Complex Detection

**Fig. 14:**
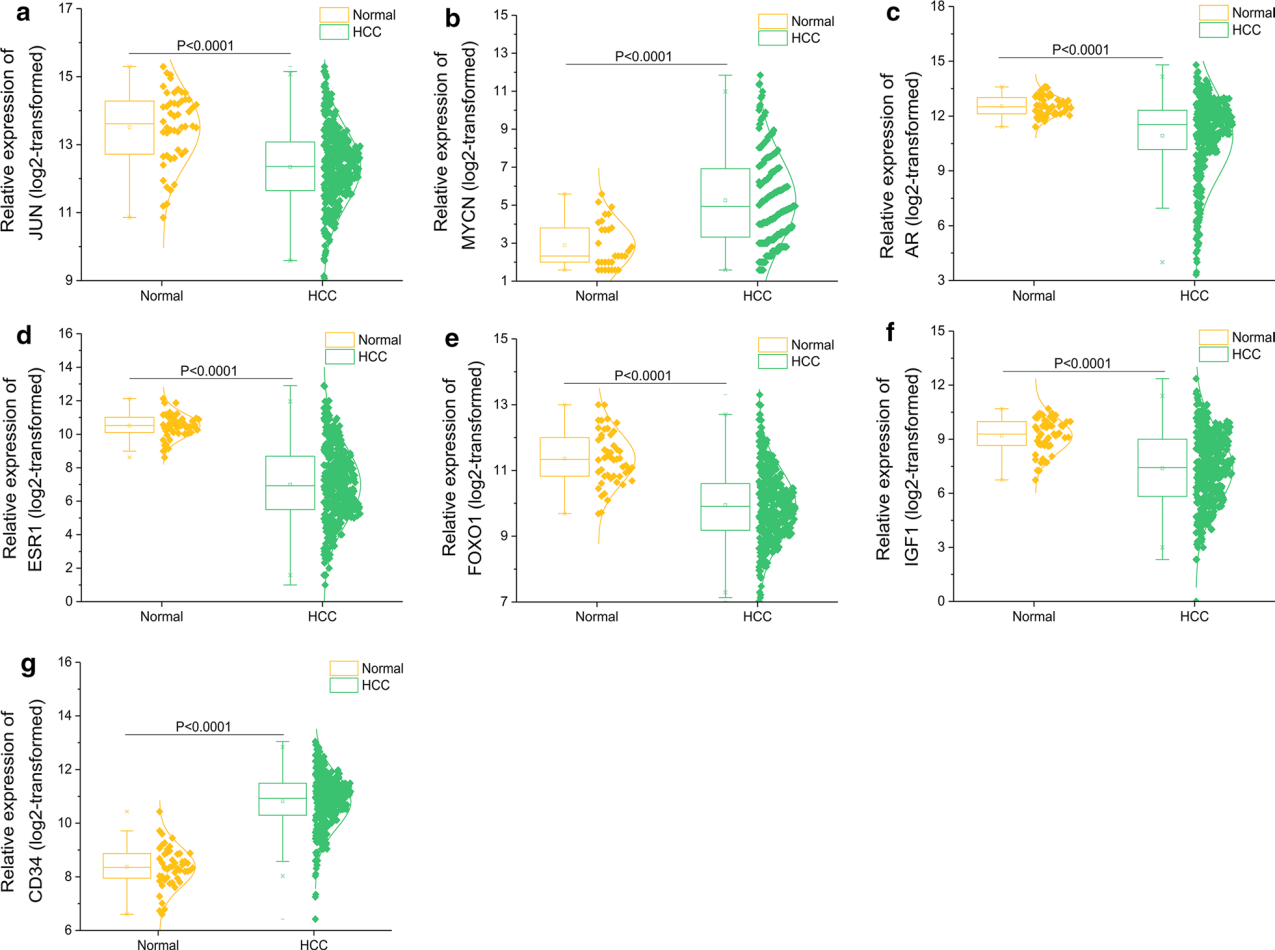
Box-scatter plots for the seven hubgenes expression in hepatocellular carcinoma (HCC): **a** JUN, **b** MYCN, **c** AR, **d** ESR1, **e** FOXO1, **f** IGF1, **g** CD34

**Fig. 15:**
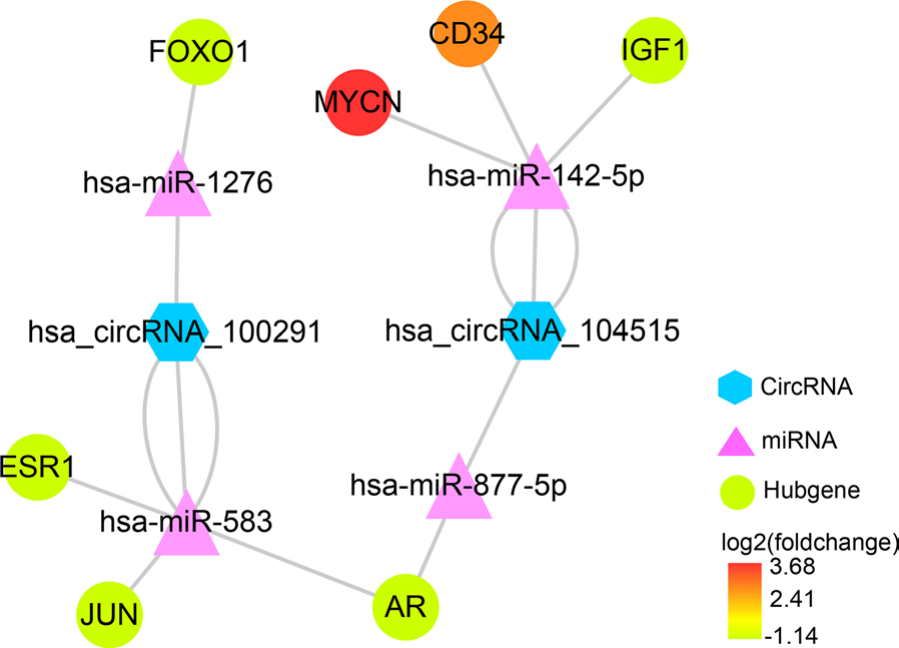
CircRNA–miRNA–hubgene network. The network consisting of two cricRNAs (hsa_circRNA_104515 and hsa_circRNA_100291), four miRNAs (hsa-miR-142-5p, hsa-miR-877-5p, hsa-miR-583 and hsa-miR-1276) and seven hubgenes (JUN, MYCN, AR, ESR1, FOXO1, IGF1 and CD34) was generated by Cytoscape 3.6.1

Removing unconnected nodes, we established a PPI network consisting of 91 nodes and 131 edges to view the interactions among the 172 mRNAs (Fig. 13a). Considering the importance of hubgene in a network, we employed a MCODE approach to screen hubgenes from the PPI network. With the k-core = 2, one subnetwork with 7 nodes and 18 edges was identified (Fig. 13b), which unveiled the critical roles of the seven genes (JUN, MYCN, AR, ESR1, FOXO1, IGF1 and CD34) in HCC. The expression levels of the seven genes in HCC are exhibited in Fig. 14. A circRNA-miRNA-hubgene network was then built to delineate the links among the DECs, miRNAs and hubgenes (Fig. 15). Eight circRNA–miRNA–mRNA regulatory modules, including hsa_circRNA_100291/hsa-miR-1276/FOXO1 regulatory axis, hsa_circRNA_100291/hsa-miR-583/ESR1 regulatory axis, hsa_circRNA_100291/hsa-miR-583/JUN regulatory axis, hsa_circRNA_100291/hsa-miR-583/AR regulatory axis, hsa_circRNA_104515/hsa-miR-877-5p/AR regulatory axis, hsa_circRNA_104515/hsa-miR-142-5p/MYCN regulatory axis, hsa_circRNA_104515/has-miR-142-5p/IGF1 regulatory axis and has_circRNA_104515/has-miR-142-5p/CD34 axis, were found from the network.

### GO annotation, KEGG pathway and Reactome pathway analyses of the seven hubgenes

**Fig. 16:**
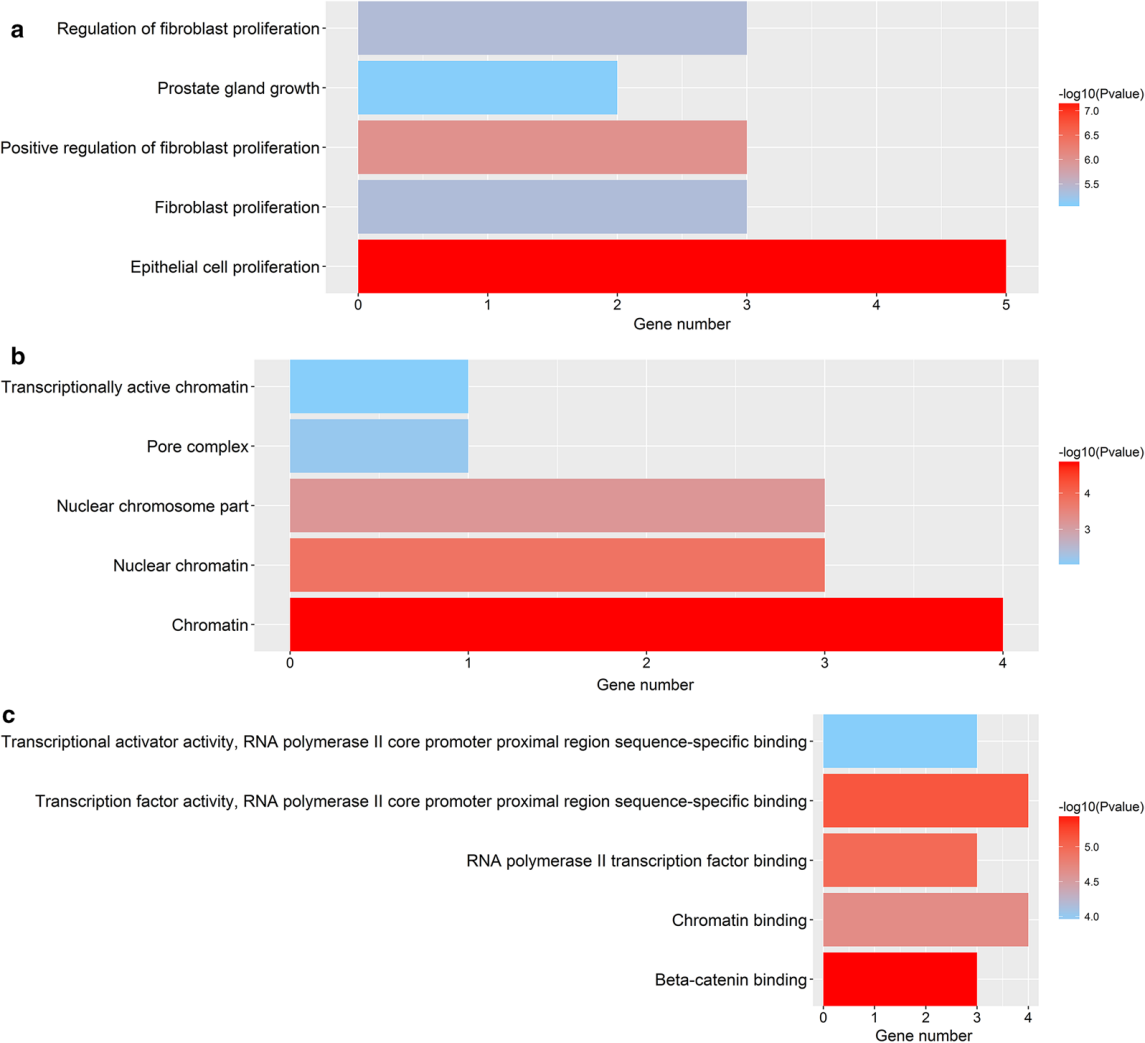
Top five Gene Ontology (GO) enrichment annotations of the seven hubgenes: **a** biological process, **b** cellular component, **c** molecular function. GO analysis was conducted by R package ‘clusterProfiler’ and visualized by R package ‘ggplot2’

**Fig. 17:**
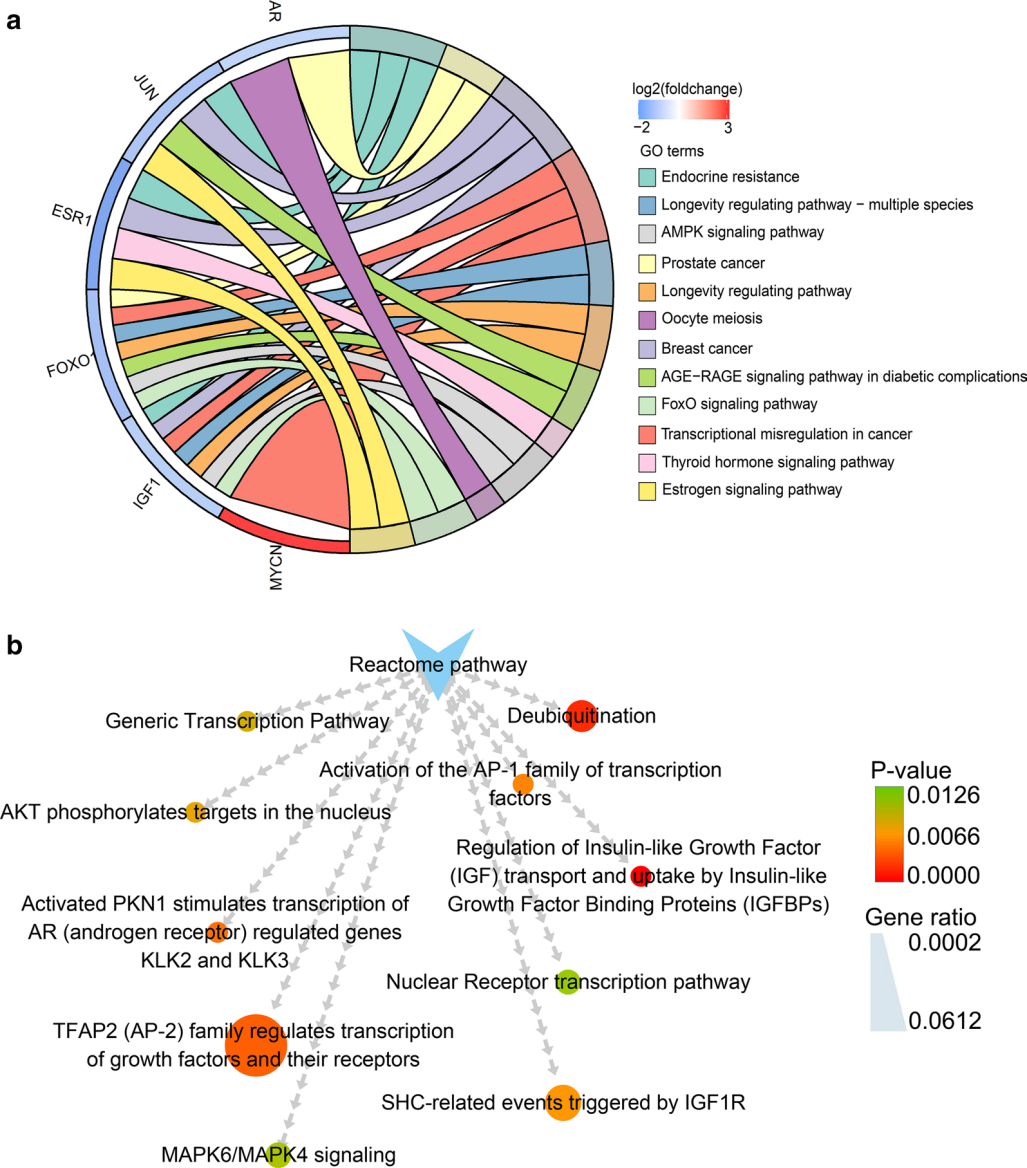
Significantly enriched Kyoto Encyclopedia of genes and genomes (KEGG) and Reactome pathways. **a** The significantly enriched KEGG pathways with an adjust P-value < 0.05. KEGG analysis was conducted by R package ‘clusterProfiler’ and visualized by R package ‘GOplot’. Cohort plot shows that the seven genes are correlated via ribbons with their assigned KEGG terms. **b** The top ten enriched Reactome pathways

GO analysis was carried out to illustrate the functional annotations of the seven hubgenes. The top five highly enriched GO terms of biological process (BP), cellular component (CC) and molecular function (MF) are shown in Fig. 16. The most enriched GO terms in BP was “epithelial cell proliferation” (P < 0.0001), that in CC was “chromatin” (P < 0.0001), and that in MF was “beta-catenin binding” (P < 0.0001). KEGG pathway analysis was conducted to ascertain the signaling cascade that the seven genes participate. With an adjust P-value < 0.05, 12 significantly enriched pathways were obtained (Fig. 17a). Among the 12 pathways, “AMPK signaling pathway”, “FoxO signaling pathway” and “Estrogen signaling pathway” are linked with the progression of HCC [27–29]. Additionally, some other pathways such as “Prostate cancer”, “Breast cancer” and “Transcriptional misregulation in cancer” were also tumor-related pathways. Reactome pathway analysis was further performed to delineate the metabolic pathways that the seven hubgenes related to. With a P-value < 0.05, a total of 29 pathways were identified (data not shown). The top 10 significantly enriched Reactome pathways are displayed in Fig. 17b.

### Identification of three bioactive compounds for the treatment of HCC based on CMap analysis

**Fig. 18:**
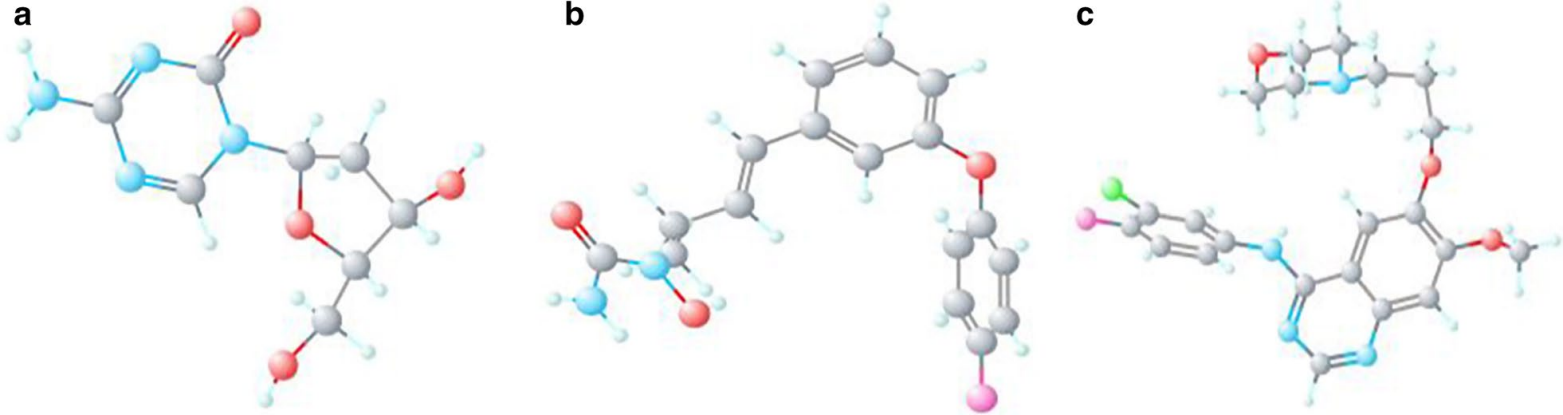
3D-structure of the three compounds identified by the connectivity map analysis: **a** decitabine, **b** BW-B70C, **c** gefitinib

The seven hubgenes consisting of two up-regulated genes (CD34, MYCN) and five down-regulated genes (AR, JUN, ESR1, FOXO1, IGF1) were loaded into the CMap web tool as up-regulated tags and down-regulated tags, respectively. Following the signature query, three compounds (decitabine, BW-B70C, gefitinib) with the highest negative enrichment score were determined as the potential therapeutic agents for HCC (Table 5). The chemical structures of the three compounds are presented in Fig. 18.

**Table 5:** Three compounds identified as treatment options for hepatocellular carcinoma by CMap analysis

CMap name	Enrichment score	Dose	Cell	Up score	Down score
Decitabine	− 0.996	100 nM	MCF7	− 0.649	0.238
BW-B70C	− 0.98	32 µM	MCF7	− 0.564	0.226
Gefitinib	− 0.979	10 µM	HL60	− 0.455	0.329

*CMap* connectivity map

## Discussion

Due to the lack of 5′ caps and 3′polyadenylated tails, circRNAs are ignored in classical polyadenylated transcriptome studies for a long time. In the past few decades, with the development of high-throughput sequencing, biochemical and computational biology methods, a large number of circRNAs are unmasked the veil in various tissues and cells [30]. Increasing study has unveiled the important roles of circRNAs in a myriad of human diseases, including malignant tumors [31–33]. CircRNAs usually serve as diagnostic and prognostic biomarkers because of their relative tolerance to exonucleases, which benefits from the covalently closed loop structures [34, 35]. In addition, owing to the high cell- and tissue- specificity of circRNAs, their roles in different neoplasms are not complete accord. In HCC, an increasing number of circRNAs, such as ciRS-7 [36], has_circ_0067934 [12], CDR1as [37] and circMTO1 [11], have been reported to exert momentous roles in regulating pathophysiological process and guiding clinical diagnosis and treatment. However, there are still a lot of circRNAs that need to be unearthed.

In this study, we firstly collected three gene chips (GSE78520, GSE94508 and GSE97332) from the GEO database and screened six DECs (hsa_circRNA_103510,hsa_circRNA_100542, hsa_circRNA_102166, hsa_circRNA_104515, hsa_circRNA_105031 and hsa_circRNA_100291) with the RobustRankAggreg approach, an algorithm for integration of different gene expression spectra, ranking genes by their fold changes and aggregating these rankings to achieve the final robust and rigorous results. Following the RT-qPCR validation of the six DECs, three circRNAs (hsa_circRNA_102166, hsa_circRNA_100291 and hsa_circRNA_104515) were selected for further analysis.

As highly conserved endogenous RNAs, many circRNAs harbors abundant miRNA binding sites, indicating that they can sponge corresponding miRNAs and thus function as ceRNAs to regulate gene expression [8, 38, 39]. To ascertain whether the aforementioned three circRNAs function as ceNAs in HCC, we predicted their MREs via two online tools, CSCD and CircInteractome. The former web tool predicted MREs within 50 bp upstream and downstream of circRNA junction point [16]. The latter web tool predicted MREs based on the TargetScane algorithm, which forecasts MREs by searching for 7mer or 8mer complementarity to the 3′ end and the seed region of each miRNA [17, 40]. We chose miRNA predicted by both algorithms as the putative target miRNA for the three circRNAs. Finally, five circRNA–miRNA interactions consisting of two circRNAs (hsa_circRNA_104515 and hsa_circRNA_100291) and five miRNAs (hsa-miR-1303, hsa-miR-142-5p, hsa-miR-877-5p, hsa-miR-583 and hsa-miR-1276) were determined. The expression of the five miRNAs was then verified based on the microarray and RNA-seq data from the GEO and TCGA. The results showed that miR-142-5p was down-regulated in HCC, which was consistent with previous studies [41, 42]. No relevant study reported the expression of miR-877, miR-1303, miR-1276 and miR-583 in HCC. According to our results, miR-877 was up-regulated in HCC. MiR-1303 and miR-1276 showed high expression tendencies in HCC, while the differences were not statistical significant. For miR-583, its expression in HCC tissues was similar to that in normal liver tissues. Due to the inter-study heterogeneity that cannot be ignored, the reliable of the pooled results is reduced. Thereby, further rigorous studies are necessary to validate these findings.

Following the collection of the 172 overlapped genes between the target genes of the five miRNAs and the DEGs in HCC, we constructed a circRNA–miRNA–mRNA regulatory network. We found that hsa_circRNA_104515 and hsa_circRNA_100291 may act as ceRNAs to capture miR-1276, miR-583, miR-877-5p or miR-142-5p, and subsequently regulate the 172 genes expression. Our results provide an evidence of the ceRNA regulatory mechanism of hsa_circRNA_104515 and hsa_circRNA_100291 in HCC. To further elucidate the action mechanism of the ceRNA network, we constructed a PPI network, screening seven hubgenes (JUN, MYCN, AR, ESR1, FOXO1, IGF1 and CD34) from the PPI network. The functional annotations and pathway analyses showed that the seven hubgenes genes were involved in many critical tumor-related biological functions and metabolic pathways, such as “epithelial cell proliferation”, “AMPK signaling pathway”, “FoxO signaling pathway”, “Estrogen signaling pathway”, “MAPK6/MAPK4 signaling” and “MAPK family signaling cascades”. The important roles of the seven genes in HCC have also been demonstrated previously [43–49]. However, their links with circRNAs have not yet been explored. Here, we identified seven circRNA–miRNA–mRNA axes (hsa_circRNA_100291/miR-1276/FOXO1, hsa_circRNA_100291/miR-583/ESR1, hsa_circRNA_100291/miR-583/JUN, hsa_circRNA_100291/miR-583/AR, hsa_circRNA_104515/miR-877-5p/AR, hsa_circRNA_104515/miR-142-5p/MYC, hsa_circRNA_104515/miR-142-5p/MYCN, hsa_circRNA_104515/miR-142-5p/IGF1 and hsa_circRNA_104515/miR-142-5p/CD34), indicating competitive regulatory relationships of hsa_circRNA_100291 and hsa_circRNA_104515 with the seven genes in HCC. However, given that the results are on the basis of computational biology, further in-depth studies are indispensable to verify the possible roles of the seven axes in HCC.

To date drug control is an important treatment for patients with HCC [50]. Digging effective and sensitive drugs against HCC helps improve patients’ outcomes. We therefore implemented the CMap analysis of the seven hubgenes to explore usable drugs for the treatment of HCC. Based on the genome-wide expression profiling of transcripts technology, CMap provides a comprehensive and accurate data resource for exploration of novel drug or relocation of existing drug [51]. Drugs available in CMap are all licensed for human use by the Food and Drug Administration, thus it is an ideal and reliable approach to obtain therapeutic agents for human diseases [52]. Three chemicals (decitabine, BW-B70C and gefitinib) were identified as the treatment options for HCC. As a cytidine antimetabolite analogue, decitabine represses DNA methylation, arresting cells into G1/S phase and inhibiting tumor cell proliferation. Its antitumor activity in solid tumors including HCC has been elucidated in previous studies [53]. Xing et al. [54] have demonstrated that decitabine could facilitate the expression of miR-122 via inhibition of methylation in HCC cells. More importantly, Skårn et al. [55] have demonstrated that miR-142 is epigenetically restrained by DNA methylation. Treating decitabine in mesenchymal cells promotes mature miR-142-5p/3p expression and thus depresses cell proliferation. We thus hypothesize that decitabine may exert its anti-HCC effect by augmenting miR-142-5p via demethylation and subsequently regulating the downstream target genes of miR-142-5p. Further well-design study is necessary to validate the conclusion. BW-B70C is an inhibitor of arachidonic acid 5-lipoxygenase. Previous study has reported its anti-neoplastic activity in leukemic cells by suppressing the NOTCH1-P13K-AKT-eNOS axis [56]. However, its anti-HCC effect and action mechanism have not been elucidated as of yet. In this study, we found its potential as therapeutic agent for HCC. More studies are needed to verify this finding. Gefitinib is a selective inhibitor of tyrosine kinase receptor used in clinic for the treatment of locally advanced or metastatic non-small cell lung cancer (NSCLC) [57]. Its antineoplastic effect on the other solid tumors including HCC has also been reported [58, 59]. However, the responsiveness of different HCC patients to gefitinib varies greatly and most patients even develop gefitinib resistance [60]. Multiple non-coding RNAs including miRNAs and lncRNAs have been reported to contribute to gefitinib resistance and sensitivity in NSCLC [61–63]. We speculate that gefitinib share the similar resistance mechanisms in HCC and lung cancer. Our study provides a theoretical basis for studying gefitinib resistance mechanism and enhancing gefitinib sensitivity in patients with HCC from the perspective of circRNA–miRNA–mRNA network.

## Conclusions

In conclusion, by employing a comprehensive strategy of big data mining, RT-qPCR and computational biology, we constructed a circRNA–miRNA–mRNA network and found that hsa_circRNA_100291 and hsa_circRNA_104515 may function as ceRNAs to exert critical roles in HCC. In addition, three bioactive chemicals (decitabine, BW-B70C and gefitinib) based on the CMap analysis was determined as therapeutic agents for HCC. Our study provides a novel insight into the pathogenesis and therapy for HCC from the circRNA–miRNA–mRNA view.

## Abbreviations

CircRNA: circular RNA
HCC: hepatocellular carcinoma
RT-qPCR: reverse transcription-quantitative polymerase chain reaction
CMap: connectivity map
ceRNA: competing endogenous RNA
MRE: miRNA response element
GEO: Gene Expression Omnibus
DEC: differently expressed circRNA
PPI: protein–protein interaction
GO: Gene Oncology
KEGG: Kyoto Encyclopedia of Genes and Genomes
cDNA: complementary DNA
CSCD: Cancer-Specific CircRNA
CircInteractome: Circular RNA Interactome
TCGA: The Cancer Genome Atlas
SMD: standard mean difference
95% CI: 95% confidence interval
RNA-seq: RNA-sequencing
MCODE: Molecular Complex Detection
DEG: differently expressed gene
BP: biological process
CC: cellular component
MF: molecular function
FDA: Food and Drug Administration
NSCLC: non-small cell lung cancer
